# A Review of ARID1A’s Role in Breast Cancer Progression: Context-Dependent Mechanisms and Therapeutic Implications

**DOI:** 10.3390/cancers18010142

**Published:** 2025-12-31

**Authors:** Gopalakrishnan Shankari, Dhamodharan Prabhu, Muthusamy Sureshan, Jeyaraman Jeyakanthan, Sundararaj Rajamanikandan

**Affiliations:** 1 Centre for Bioinformatics, Karpagam Academy of Higher Education, Coimbatore 641021, India; 2 Department of Biochemistry, Karpagam Academy of Higher Education, Coimbatore 641021, India; 3 Department of Biotechnology, Karpagam Academy of Higher Education, Coimbatore 641021, India; 4 Department of Biotechnology, Faculty of Engineering, Karpagam Academy of Higher Education, Coimbatore 641021, India; 5 Department of Bioinformatics, Alagappa University, Karaikudi 630003, India; 6 School of Pharmaceutical Science and Technology, Tianjin University, Tianjin 300072, China

**Keywords:** SWI/SNF chromatin remodeling complex, ARID1A, TNBC, tumor progression, biomarker

## Abstract

ARID1A, a key subunit of the SWI/SNF chromatin remodeling complex, plays a context-dependent function in cancer, acting both as a tumor suppressor and, in certain conditions, as an oncogene. ARID1A, as a tumor suppressor, maintains transcriptional regulation, genomic stability, and cellular differentiation. In breast cancer, ARID1A loss-of-function leads to dysregulation of cell cycle checkpoints and impaired DNA repair and promotes epithelial-to-mesenchymal transition (EMT), jointly accelerating tumor proliferation and increasing therapeutic resistance. Notably, context-dependent ARID1A loss-of-function often concurs with activation of the PI3K/AKT signaling pathway and corresponds with poor prognosis. On the contrary, aberrant ARID1A overexpression can provoke oxidative stress and agitate the cytochrome P450 system, potentially facilitating early tumorigenesis. Consequently, understanding ARID1A’s dual and context-dependent role highlights its potential as a biomarker and therapeutic target in precision oncology.

## Simple Summary

Around 2.3 million new breast cancer cases arise every year globally. Early diagnosis and targeted therapy are important to developing new therapeutic drugs for breast cancer treatment. This study aims to understand the context-dependent role of ARID1A, a key component of the SWI/SNF chromatin remodeling complex that shows frequent alterations across cancers, including breast cancer. Understanding ARID1A’s role is important because loss of ARID1A disrupts DNA repair, cell-cycle control, and chromatin regulation, leading to aggressive tumor progression via enhancing EMT and activating PI3K/AKT oncogenic signaling; conversely, abnormal overexpression of ARID1A may induce oxidative stress by CYP450 and initiate tumor growth. Thus, this study highlights the context-dependent role of ARID1A and supports its potential as a diagnostic biomarker and therapeutic target for the treatment management of breast cancer and other malignancies.

## 1. Introduction

Among malignancies, metastasis remains a major cause, placing a financial and psychological burden on affected individuals. During metastatic progression, genomic alterations underscore the importance of identifying genetic markers to inform therapeutic strategies and improve cancer management. In the early stages, clinically significant genetic mutations in cancers are prevalent and complex [1].

Globally, breast cancer is the second most common cancer and remains the most common cancer in women. WHO reports that about 2.3 million new cases are diagnosed every year [https://www.who.int/news-room/fact-sheets/detail/breast-cancer; accessed on 14 August 2025]. In 2022, it was the leading cancer diagnosis in 157 of 185 countries and caused 670,000 deaths. As stated by the International Agency for Research on Cancer (IARC), about 1 in 20 women worldwide will develop breast cancer during their lifetime [https://www.iarc.who.int/news-events/breast-cancer-cases-and-deaths-are-projected-to-rise-globally; accessed on 24 February 2025]. The agency projects that if this continues every year, by 2050, the number of new breast cancer cases might increase to 3.2 million, with 1.1 million deaths every year [2,3]. Among all breast cancer cases, TNBC accounts for approximately 10–20% and is more widespread among women [4,5]. In India, breast cancer accounts for 13.5% of all cancers and shows a rising trend among younger women. It is projected to become the leading cause of cancer-related deaths among Indian women by 2030 [6]. The disease continues to rise in occurrence; India bears almost one-third of the worldwide burden, resulting in over 70,000 deaths annually [7]. Over the past four decades, the occurrence of breast cancer has progressively increased, affecting 19 out of every 100,000 Indian women. A large cohort study involving 8654 complex breast malignancy samples revealed that 80.4% of malignancies had genetic alterations in at least one clinically significant pathway [8].

Breast cancer is a major global public health problem for women. The prevalence and incidence rates of breast malignancy have risen significantly over the past several years. Triple-negative breast cancer (TNBC) is a heterogeneous and aggressive subtype of breast cancer that lacks the expression of estrogen receptors (ERs), progesterone receptors (PRs), and human epidermal growth factor receptor 2 (HER2). Compared to other breast cancer subtypes, TNBC is associated with a higher incidence of metastasis, poor overall survival rates, and greater treatment complexity. Although several molecular targets, like phosphatidylinositol 3 kinase/protein kinase B/mammalian target of rapamycin (PI3K/AKT/mTOR), have been explored for TNBC therapy, their efficacy remains limited [9].

Recent clinical trials have significantly advanced breast cancer treatment across disease subtypes. The KEYNOTE-522 enrolled 1174 patients with stage II–III triple-negative breast cancer (TNBC), demonstrating clear benefit from adding pembrolizumab to standard therapy [10]. In the case study of IMpassion130, which included 902 patients with advanced or metastatic TNBC, improved outcomes were observed with atezolizumab in PD-L1–positive disease [11]. The large TAILORx trial (NCT00310180) followed 10,273 women with early-stage, HR^+^/HER2^-^ breast cancer, helping refine which patients truly need chemotherapy. Similarly, monarchE studied 5637 women with high-risk HR^+^/HER2^-^ early breast cancer, supporting the use of adjuvant abemaciclib [12]. In CREATE-X, 910 HER2^-^ patients with residual disease after neoadjuvant chemotherapy showed improved survival with adjuvant capecitabine [13,14]. Even though major progress has been made in large clinical trials like KEYNOTE-522, IMpassion130, TAILORx, monarchE, and CREATE-X, breast cancer continues to be a significant health burden. Collectively, these studies have helped to enrich treatment across various subtypes and significantly expanded therapeutic options for many patients. This highlights the need for continued research for better biomarkers, more effective targeted therapies, and treatment strategies that can further improve outcomes for all patients.

Together with this background, our objective is to investigate the crucial role of ARID1A, a key chromatin-remodeling gene whose alterations are increasingly linked to breast cancer and several other malignancies. Understanding its functional impact may provide new insights into tumor development, therapeutic vulnerabilities, and potential targets for precision cancer treatment.

## 2. SWItch/Sucrose Non-Fermentable (SWI/SNF) Chromatin Remodeling Complex

An epigenetic modification is an important early event in cancer and contributes to frequent changes in the functioning of cancer cells. Multitude evidences indicates that the SWI/SNF (Switch/Sucrose Non-Fermentable) chromatin remodeling complex is important for the regulation of genomic integrity, transcription, and the development of cellular components [15]. Targeting the SWI/SNF complex may provide therapeutic strategies to exploit the vulnerabilities associated with its divergences in malignancies.

The ATPase subunits of the SWI/SNF chromatin remodeling complex represent a group of evolutionarily conserved proteins responsible for regulating chromatin accessibility, relocating nucleosomes through ATP hydrolysis. The bromodomain in these ATPase subunits aids in the recognition of acetylated histones [16]. Functionally, these complexes are important for regulating gene expression and maintaining stem cell pluripotency. Three distinct forms of SWI/SNF complex are BRG1/BRM-associated factor (BAF), Polybromo-associated BAF complex (PBAF), and non-canonical BAF (ncBAF) complex. Each of these has unique subunits that assemble in various forms to modulate nucleosome positioning and chromatin accessibility. Via these mechanisms, the SWI/SNF complex plays a crucial role in DNA repair, replication, and transcriptional initiation [17]. The interplay among the SWI/SNF complex and other epigenetic regulators boosts the impact on gene expression. This complex works with histone modifiers, histone acetyltransferases (HATs), and histone deacetylases (HDACs) to produce either a permissive or restrictive chromatin state and interacts with DNA methyltransferases (DNMTs) and ten-eleven translocation (TET) enzymes to alter DNA methylation patterns. Thus, the SWI/SNF complex functions as an epigenetic integrator that dynamically regulates chromatin architecture corresponding to various physiological signals [18]. Around 20–25% of all malignancies have mutations in the SWI/SNF chromatin remodeling complex and underscore its important role in tumor proliferation. These genetic alterations reduce the expression of SWI/SNF subunits by interrupting regular chromatin remodeling and transcriptional regulation [19].

## 3. Dysregulation of SWI/SNF in ER^+^ Cancer

Genetic mutations, deletions, and aberrant expression of the SWI/SNF complex’s subunits often result in its dysfunction in breast cancer. This affects and disturbs chromatin modifications and genome stability by promoting the growth of tumor cells. These changes alter the role of estrogen receptor (ER), which plays a major role in hormone receptor-positive breast cancer [20]. The SWI/SNF complex regulates gene expression by enabling the binding of ER to its target genes in estrogen receptor-positive (ER^+^) breast cancer. ER binds to estrogen, interacts with specific DNA sequences, and activates genes implicated in cell growth and proliferation. The functional impairment mechanism of the SWI/SNF complex becomes disturbed, which leads to abnormal gene expression and enhanced tumor progression. Moreover, dysregulation of the SWI/SNF complex also provides resistance against endocrine therapies, such as tamoxifen, by inhibiting ER activation. The malfunctioning SWI/SNF complex allows cancer cells to bypass estrogen signaling or activate substitute pathways, thereby decreasing the therapeutic efficacy of treatment [21,22]. Furthermore, SWI/SNF dysfunction disturbs the stimulation of epithelial-to-mesenchymal transition (EMT), a critical mechanism in metastasis. EMT is a regulated process in which epithelial cells acquire mesenchymal traits, including increased motility, invasiveness, and resistance to apoptosis. It is largely driven by microenvironmental signals such as TGF-β, Wnt, and Notch. Though genetic alterations might affect cells to undergo EMT, external signals from the tumor microenvironment mostly begin and maintain this process. Thus, this perspective highlights the regulation of EMT transitions [23,24]. This complex activates transcription factors that restrain epithelial markers such as E-cadherin and upregulate mesenchymal markers such as vimentin, thus enhancing cell invasion and motility [25,26]. Therefore, the dysregulation of the SWI/SNF complex has important implications for clinical results and metastatic progression in ER^+^ breast cancers.

## 4. ARID1A: Biological Functions and Its Role in Breast Cancer

ARID1A (AT-rich interaction domain 1A) is a subunit of the canonical BAF (cBAF) in the SWI/SNF chromatin remodeling complex that controls where the complex binds regulatory DNA in differentiated cells. Its crucial role is maintain genomic integrity, as shown by its frequent mutations in various cancers, including gastric and ovarian cancers [27,28]. ARID1A is important for targeting SWI/SNF to genomic spots occupied by transcription factors, such as estrogen receptor (ER), GATA3, and FOXA1 in luminal breast epithelial cells. Mutations or deficiency of ARID1A disrupt this mechanism and cause SWI/SNF to fail to reside in cis-regulatory elements [29,30]. The ARID1A protein performs its primary function across different cancers by positioning cBAF at tissue-specific regulatory elements [27,30]. Loss of ARID1A drives a luminal-to-basal lineage modification in ER^+^ breast cancer, which decreases ER^-^ dependent transcription and resistance to endocrine therapies such as fulvestrant by separating SWI/SNF from ER/GATA3/FOXA1-bound enhancers [28,29,31]. By engaging HDAC1 to suppress histone H4 acetylation, ARID1A acts as a controller; therefore, ARID1A deficiency results in increased H4 acetylation and activates growth-promoting transcriptional programs [27,32]. ARID1A resists ER^-^ driven proliferative signaling and tumor growth in endometrial cancer. This indicates that ARID1A limits ER production in a tissue-specific context [33,34]. Linking of ARID1A to androgen receptors (ARs) has been scarcely examined in recent studies; ARID1A modifies steroid receptor-linked lineage programs, suggesting potential for corresponding interactions [35]. AR shows context-dependent roles in ER breast cancers that loss of ARID1A disrupts chromatin regulators affecting drug sensitivities and self-regulation of ER; thus, future investigation of ARID1A-AR interactions across breast cancer subtypes is necessary [27,32]. ARID1A, since it is a tumor suppressor, experiences weakened functions due to loss of SWI/SNF targeting to lineage-specific enhancers, leading to dedifferentiation and therapy resistance. Reduced HDAC1 binding increases acetylation-dependent transcription of oncogenic programs, and ARID1A mutations yield distinct phenotypes depending on tissue-specific transcriptional changes [27,30,36]. Thus, understanding the role of ARID1A opens a new path for advanced treatment approaches in cancer biology. The restoration of wild-type ARID1A expression shows suppressed cell proliferation and tumor growth, highlighting its potential as a therapeutic target in ARID1A-mutated cancers [33]. This developing understanding of ARID1A’s role in breast cancer not only highlights its implications for treatment but also broadens the landscape of cancer biology. By explaining the mechanisms through which ARID1A mutations influence tumor behavior and immune interactions, researchers can address unique characteristics of both ER^+^ and ER^-^ breast cancers, enhancing the precision of oncology and improving patient outcomes.

The significant cross-talk between ARID1A and genomic instability in TNBC is due to the loss of ARID1A, which disrupts DNA damage repair and increases the accumulation of genomic alterations. ARID1A is lost or downregulated (mutation, deletion, epigenetic silencing) in a subset of TNBC tumors [37]. In the TNBC context, genomic instability might fuel the aggressive phenotype: high metastatic potential, poor prognosis, and possibly contribute to immune evasion/immunoediting. Indeed, TNBC tumors with low ARID1A show poor outcomes [9,38,39]. Additionally, genomic instability may make such tumors more immunogenic (neoantigens), potentially affecting immune infiltration and response to immunotherapy, which is especially relevant since some TNBCs are treated with immune checkpoint inhibitors. The general role of ARID1A loss in activation of the cytosolic DNA-sensing (cGAS–STING) pathway after DNA damage supports this possibility [37,39].

Although extensive research has established that ARID1A regulates DNA double-strand break repair, replication stress responses, and chromatin structure in other tumor models, comparable mechanistic validation in TNBC models is still lacking [37,40].

## 5. Correlation of ARID1A and PIK3CA in TNBC and Other Cancers

ARID1A (AT-rich interactive domain-containing protein 1A), a critical component of the SWI/SNF remodeling complex, is located on chromosome 1p36.11. It encodes the protein BAF250a, which plays a crucial role in regulating gene expression through chromatin structure modification. Mutations in this regulatory gene have been identified in various human cancers, including breast cancer [41,42]. Co-occurring ARID1A deficiency and PI3KA mutations are observed in ~6% of all cancer cases [43].

### Functional Consequences of ARID1A in Tumor Progression

Previous studies have shown that loss of ARID1A leads to the activation of several oncogenic pathways, including the PI3K/AKT pathway, nuclear localization of YAP (Yes-Associated Protein), and Hippo pathway, thereby enhancing cell proliferation and metastatic potential in TNBC cells. Recent studies further demonstrate that the loss of ARID1A expression increases EMT (epithelial–mesenchymal transition) markers’ expression, which are associated with enhanced migration and tumor cell aggression [9,44]. Under normal circumstances, ARID1A-proficient cells sustain proliferation homeostasis, as ARID1A negatively regulates the PI3K/AKT signaling pathway. However, loss-of-function or mutation of ARID1A disturbs this balance, resulting in abnormal activation of PI3K/AKT signaling. This dysregulation has been implicated in various malignancies, including TNBC, and is frequently associated with poor diagnosis and violent tumor behavior [45,46,47,48]. The interplay between ARID1A and the PI3K/AKT pathway plays a significant role in tumor development. In TNBC, the PI3K/AKT pathway is often dysregulated, and signaling pathways are activated through genetic mutations or loss of PTEN, thus promoting tumorigenesis and therapeutic resistance [49]. Around 60% of TNBC patients show PTEN alterations, which activate the PI3K/AKT pathway and increase tumor cell growth and survival.

Targeting of the PI3K/AKT pathway, therefore, has received significant therapeutic interest. However, the development of resistance to inhibitors such as buparlisib and MK-2206 has compromised their clinical effectiveness. Preclinical studies suggested a dual therapeutic strategy inhibiting the PI3K/AKT signaling pathway while restoring ARID1A and PTEN expression, which may be used to overcome and conquer resistance and develop treatment outcomes [50]. Drugs able to reactivate ARID1A and PTEN expression, by inhibiting PI3K/AKT and YAP signaling, are likely to suppress tumor growth and increase survival in TNBC patients [51]. Additionally, re-expression of ARID1A reduces cell proliferation, confirming its role as a tumor suppressor and ability to inhibit PI3K/AKT pathway activation [52].

## 6. Clinical and Preclinical Evidence for Targeting ARID1A and PIK3CA

Approximately 33% of ovarian clear cell carcinoma cases exhibit co-mutations of ARID1A and PIK3CA, which activate pro-inflammatory cytokine genes via the NF-kB pathway, thereby promoting tumor growth. In an in vivo experiment using engineered mice harboring ARID1A loss and PIK3CA mutations, treatment with the PIK3 inhibitor buparlisib led to a dose-dependent reduction in AKT levels and tumor viability, highlighting the therapeutic potential of buparlisib in OCCC with these co-mutations [32]. Similarly, a clinical trial with PI3K inhibitor copanlisib in PIK3CA- and ARID1A-mutant cancers demonstrated a significant decrease in tumor growth. The inhibition mechanism was attributed to PUMA induction mediated by FOXO3a, which was aberrantly expressed following p53/p21 pathway dysregulation caused by ARID1A loss [53]. Similarly, a high frequency of cholangiocarcinoma cases also harbor ARID1A and PI3K mutations. Studies evaluating AKT inhibitors (MK-2206) in ARID1A-deficient cholangiocarcinoma cell lines revealed increased sensitivity to the drug, suggesting that targeting the PI3K/AKT pathway could improve therapeutic outcomes in these tumors [54].

Using ARID1A knockdown in cell-based assay and mutant PIK3CA human ovarian epithelial cell lines, the grouping of mutations induced cell transformation and cytokine gene induction. Systematically, ARID1A loss-of-function enables the recruitment of the Sin3A-HDAC complex, while PIK3CA mutations promote the release of RelA from IkB, both of which are most important for cytokine gene activation. These findings suggest that NF-kB inhibition may represent a viable therapeutic strategy for tumors harboring these co-mutations [55].

Furthermore, when using in vivo models of ARID1A- and PIK3CA-mutated ovarian clear cell carcinoma, treatment with an HDAC6 inhibitor reduced tumor growth and improved survival rates by promoting the activation of IFN gamma-positive CD8 T cells. For both in vitro and in vivo studies using patient-derived xenografts from ARID1A-deficient bladder cancer cell lines, treatment with the EZH2 inhibitor GSK-126 exhibited synergistic effects in reducing cell viability, highlighting the potential of PI3K pathway inhibitors in ARID1A-deficient bladder cancer therapy [56].

Additionally, inhibition of the PI3K pathway using LY294002 in ARID1A-deficient, radioresistant pancreatic cancer cells increased apoptosis and impaired DNA damage repair, further supporting the therapeutic relevance of this approach [57,58]. A large-scale screening of 551 kinase inhibitors in ARID1A isogenic gastric cancer cells identified the AKT inhibitor AD5363 (capivasertib) as particularly effective, significantly reducing cell viability by activating the Caspace-3/GSDME pathway. Collectively, these studies underline the importance of PI3K/AKT signaling in cancers with ARID1A deficiency and PIK3CA co-mutations [59]. A study of the molecular mechanism underlying ARID1A’s role in cancer development, with prognostic implications and pathological uniqueness, remains unexplored [48]. Ongoing research focusing on ARID1A and PI3K/AKT for the treatment of TNBC, by restoring ARID1A expression and inhibiting PI3K/AKT signaling, could successfully reduce tumor aggressiveness and improve the clinical outcomes in TNBC.

## 7. Context-Dependent Role of ARID1A

By enabling DNA repair, regulating cell cycle checkpoints, and maintaining epigenetic stability, ARID1A acts as a tumor suppressor. Loss of function of ARID1A results in genomic instability, accumulation of mutations, and tumor initiation with uncontrolled cell proliferation [30]. In later stages of tumorigenesis, haploinsufficiency or complete loss-of-function ARID1A is linked to enhanced tumor development, particularly by promoting cell migration and metastasis. This is mediated by the downregulation of tumor-suppressor genes such as E-cadherin, along with transcriptional reprogramming toward metastasis-related gene expression profiles [60,61,62]. E-cadherin, encoded by the CDH1 gene, is a traditional tumor suppressor that upholds epithelial integrity by promoting robust calcium-dependent cell–cell adhesion. The epithelial–mesenchymal transition (EMT) is a critical stage in tumor invasion and metastasis, disturbing tissue construction and cell polarity by promoting loss of E-cadherin. Loss of E-cadherin is associated with the development of human cancers. In many epithelial cancers, inactivation of CDH1 occurs via mutations, promoter hypermethylation, transcriptional repression, or proteolytic cleavage [63,64,65]. This finding underscores the crucial role of ARID1A deficiency in chromatin architecture and gene regulation in facilitating metastatic activity.

## 8. Underlying Mechanisms of ARID1A in Oncogenic Functions

Current studies and findings show that the role of ARID1A is highly context-dependent, acting as both a tumor suppressor and a tumor promoter depending on the cancer stage, tissue type, and mutational background [30,62,66]. Depending on the environment and ecological factors, a gene can play a role in cancer cells, either promoting or suppressing tumors. This point of view is reinforced by current findings that the role of ARID1A is highly context-dependent, varying with cancer stage and mutations. As a result, this study seeks to accept the tumor system view and tumor ecology and to make a fundamental break from the theoretical constraints of the existing somatic mutation paradigm. The researchers expect that cancer research will move toward ecological landscapes, forming a new system different from genetic molecular maps [67]. In various mouse models of liver cancer, ARID1A has been shown to exhibit tumor-promoting activity by promoting early transformation, potentially through regulation of oxidative stress pathways mediated by the cytochrome P450 (CYP450) system, which generates reactive oxygen species (ROS). High ROS levels provoke DNA damage, promote mutagenesis, and create a tumor-permissive background that accelerates both initiation and progression in liver cancer.

ARID1A overexpression impacts long-range chromatin modifications that enable transcription factors, such as SOX6, to reinforce the expression of oncogenic genes. This collaboration enhances gene expression and promotes cell propagation and migration while repressing tumor suppressor genes involved in cell adhesion, like E-cadherin, thereby facilitating invasiveness and metastasis [68,69]. ARID1A overexpression and increased oxidative stress align with established studies indicates that antioxidants can suppress tumor initiation but might, in contradiction, promote tumor progression when redox balance is disrupted [70,71]. Moreover, ARID1A alters the activity of transcription factors like HNF4A that regulate hepatocyte differentiation, leading to dedifferentiation and a more stem-like, aggressive phenotype. ARID1A overexpression can alter epigenetic regulation, favoring a pro-oncogenic state by upregulating cell cycle regulators through activation of E2F target pathways.

In fact, loss of ARID1A might enhance oncogenic signaling, such as PI3K/AKT activation, leading to tumor growth. The absence of ARID1A compromises DNA repair pathways, especially homologous recombination, leading to genomic instability. The instability that promotes or inhibits tumor development appears to depend on the stage of malignancy. A comprehensive study indicates that in the early stages of carcinogenesis, ARID1A acts as an impediment to transformation, maintaining chromatin integrity and genomic stability. As tumors develop, mutational inactivation of ARID1A enables cancer cells to bypass growth limitations and adjust to an active tumor microenvironment.

## 9. Therapeutic Strategies and Resistance Mechanisms

### 9.1. EZH2 Inhibitors

ARID1A-mutant cancers are particularly sensitive to EZH2 inhibition (e.g., GSK126), an effect linked to the suppression of PI3K/AKT signaling. This has led to clinical evaluation of EZH2 inhibitors, such as tazemetostat, in tumors harboring ARID1A alterations. However, resistance to EZH2 inhibitors can arise through mechanisms such as switching from SMARCA4 to SMARCA2 or upregulation of BCL2, both of which represent potential targets to overcome therapeutic resistance [31].

### 9.2. PARP Inhibitors

ARID1A deficiency compromises double-strand break repair and impairs DNA damage checkpoint signaling, providing a mechanistic rationale for the sensitivity of these tumors to PARP inhibitors (PARPi) in preclinical models. However, responses to PARPi are heterogeneous and can be limited by established resistance mechanisms, including restoration of homologous recombination, PARP1 downregulation, and enhanced replication fork protection [40].

### 9.3. PI3K/AKT/mTOR Inhibitors

**Figure 1:**
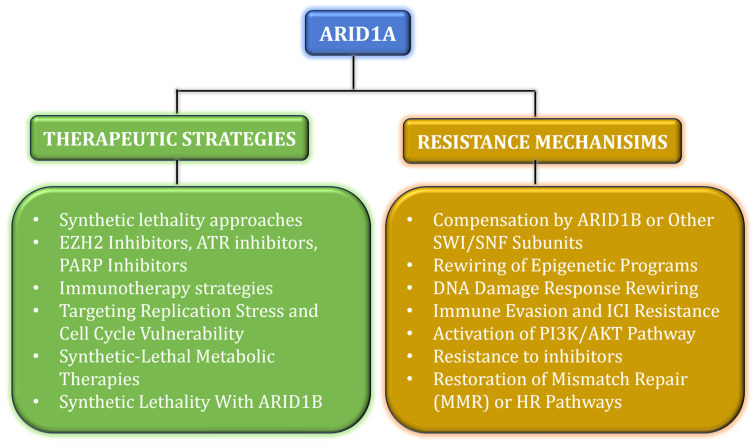
The schematic representation shows the therapeutic approaches in green and the resistant mechanisms in mustard for ARID1A.

ARID1A-deficient tumors can become PI3K/AKT-dependent via upregulation of the phosphatidylinositol 3-kinase regulatory subunit (PIK3R3). These tumors are particularly sensitive to PI3K inhibitors, such as alpelisib and pictilisib, especially when used in combination with EZH2 inhibitors. These findings provide a strong rationale for evaluating PI3K pathway inhibitors in preclinical and clinical studies targeting ARID1A-altered tumors [57]. Table 1 shows the mechanism of drugs that target ARID1A; Figure 1 displays the therapeutic approaches and resistant mechanisms of ARID1A.

### 9.4. Other Strategies

Several therapeutic strategies exploit synthetic lethality in ARID1A-deficient tumors, including ATR inhibitors, HDAC inhibitors, PLK1 inhibitors, BCL2 inhibitors, and other targeted agents. Additionally, immune-modulating strategies are under investigation, as ARID1A loss can increase neoantigen load and induce DNA mismatch repair (MMR) deficiencies in certain tumors, potentially enhancing responsiveness to immunotherapy [43,85].

Current multi-omics studies indicate that ARID1A loss-of-function drives adaptive transcriptional reprogramming that grants resistance to targeted therapies. For instance, ARID1A-deficient cells maintain MAPK1/3 and JNK activity, enabling evasion of BRAF/MAPK inhibition, and improved PI3K/AKT signaling can bypass the effects of overcoming targeted treatments, highlighting the need for combination or context-specific therapeutic approaches [86,87]. Weakened PARP inhibitor effectiveness includes the restoration of HR [88]. Efflux resistance enhances the expression of drug efflux pumps, modifies drug metabolism through CYP450 modulation [43], and creates changes in histone modification patterns that alter transcriptional accessibility [79].

### 9.5. Combination with PARP Inhibitors—Included Therapies

Preclinical and translational studies support combining PARP inhibitors with DNA-damaging agents and other pathway inhibitors to exploit ARID1A deficiency, as tumors lacking ARID1A exhibit enhanced PARPi sensitivity. Loss of ARID1A disturbs homologous recombination and cell cycle checkpoints, increasing reliance on PARP-mediated DNA repair and enabling synthetic lethality with PARP inhibitors either as monotherapy or in combination with other agents [74,76].

PI3K pathway inhibition can suppress HR repair, thereby sensitizing HR-proficient or ARID1A-altered tumors to PARP inhibitors. Preclinical models and clinical reports support the combination of olaparib with PI3K inhibitors, such as alpelisib, as an effective strategy in tumors devoid of canonical HR defects. This underscores the potential therapeutic applicability of PARPi [75,80].

Additionally, Ataxia-Telangiectasia and Rad3-related protein (ATR) inhibitors have been recognized as synthetic lethal partners for ARID1A-deficient tumors and represent rational combinations with PARPi. Preclinical reports of BRCA-related models indicate that ATR and PARPi co-treatment can overcome homologous recombination restoration and PARPi resistance, making this approach promising for targeting ARID1A-deficient cancers [75,77,82].

## 10. Stage-Specific Impact of Tumor Development

The bidirectional function of ARID1A highlights its complex role in tumorigenesis, which is influenced by factors such as timing, expression levels, and cellular context. Understanding its role is important for identifying therapeutic treatments to restore ARID1A expression [30,89]. The molecular mechanisms indicate that ARID1A overexpression can act as a vital driver of tumor initiation and progression by altering chromatin remodeling, metabolic regulation, and transcriptional reprogramming.

## 11. Conclusions

**Figure 2:**
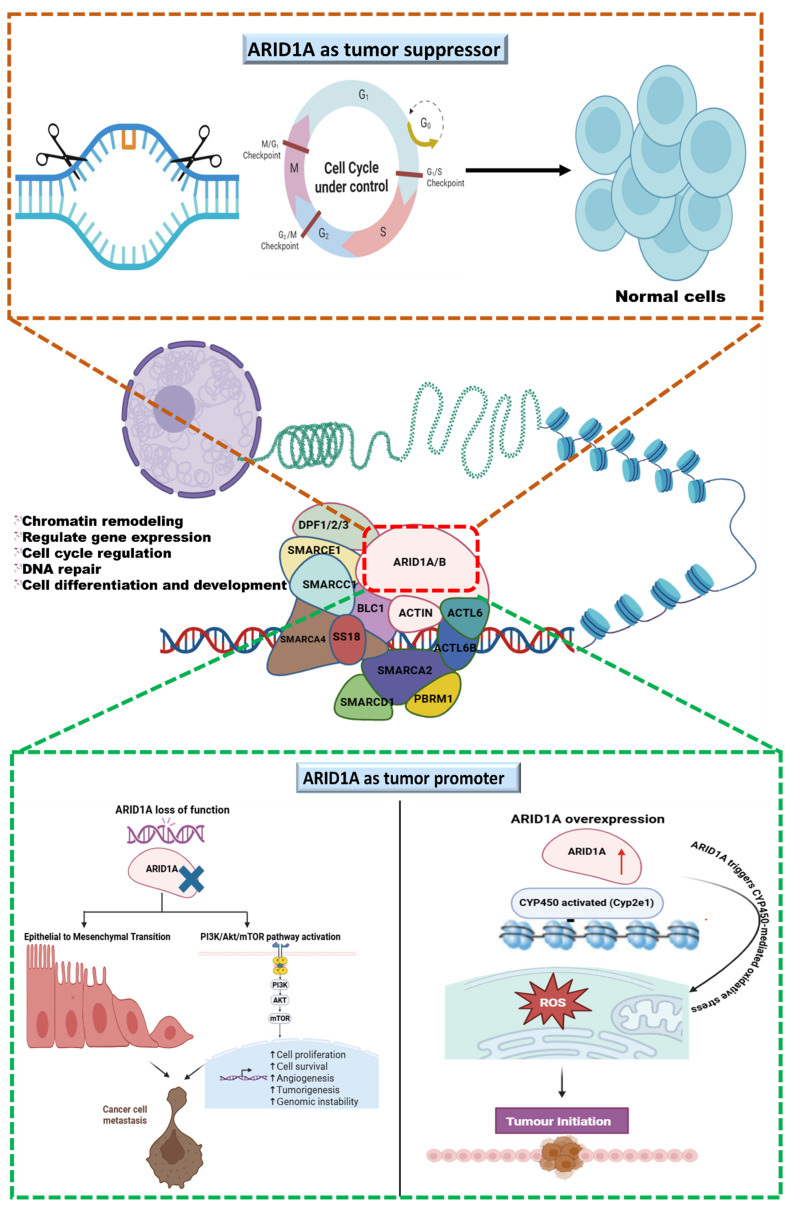
The graphical illustration represents ARID1A as a tumor suppressor, a key subunit of SWI/SNF remodeling complex that maintains DNA repair, and ARID1A’s role in genome regulation and context-dependent consequences. ARID1A as a tumor suppressor controls cell cycle and maintains genome stability, whereas at the bottom, featuring ARID1A as a tumor promoter, the left panel shows that ARID1A’s divergent dysregulation loss of function promotes oncogenesis through EMT and PI3K/AKT/mTOR activation, leading to cancer cell metastasis; the right panel shows that ARID1A’s overexpression promotes oncogenesis via CYP450-mediated ROS generation and initiates the tumor.

ARID1A is an ambiguous regulator in cancer biology, as its role in triple-negative breast cancer (TNBC) is context-dependent. Usually, it functions as a tumor suppressor by maintaining chromatin structure, regulating genomic stability, and supporting DNA repair. However, ARID1A exhibits oncogenic features under specific cellular circumstances. In TNBC, loss-of-function mutations or downregulation of ARID1A disturb cell cycle regulation and DNA repair, increase EMT, and activate the PI3K/AKT pathway, forcing aggressive tumor proliferation and resistance to treatments. Conversely, overexpression of ARID1A deregulates oxidative stress and promotes early tumorigenesis and metastasis by altering transcriptional programs (Figure 2). The frequent co-occurrence of ARID1A loss with PIK3CA mutations in various cancers, including TNBC, ovarian clear cell carcinoma, and cholangiocarcinoma, highlights the vital role of ARID1A and PI3K/AKT in tumorigenesis. This provides a strong rationale for targeting ARID1A in combination with PI3K/AKT signaling.

Thus, ARID1A’s dualistic function highlights the need for context-specific treatment approaches. Continuous research, along with precision oncology and biomarker-driven clinical trials, makes ARID1A a promising therapeutic biomarker in all malignancies and also improves TNBC treatment management.

## Figures and Tables

**Table 1:** Therapeutic drugs targeting ARID1A showing drug mechanisms, resistance, and current status of drugs in clinical development.

S. No.	Drugs	Mechanism	Resistant	In ARID1A Models	Status	References
1	Olaparib	PARP inhibitors	PARPi resistance via HR restoration, replication fork stabilization, reduced PARP trapping	Trap PARP on damaged DNA and exploit defective HR seen with ARID1A loss. ARID1A-deficient CRC cells and preclinical models show increased PARPi sensitivity and temozolomide sensitizivity of ARID1A-mutant models to PARPi	FDA approved for multiple HR-deficient indications; for BRCA-mutated breast cancer; for ovarian cancer maintenance; preclinical ARID1A studies	[72,73,74,75,76]
2	Niraparib					
3	Rucaparib					
4	Talazoparib					
5	Temozolomide					
5	GSK126	EZH2 inhibitors	Potential for epigenetic adaptation; pair with PI3K for synergy	EZH2 inhibition shows selective activity in ARID1A-deficient gastric cancer models in preclinical studies, via modulation of PI3K/AKT and chromatin states	FDA approved for epithelioid sarcoma; synthetic lethal with ARID1A loss	[77,78,79]
6	Tazemetostat					
7	Alpelisib	PI3Kα inhibitors, Pan-class I PI3K inhibitors, dual PI3K/mTOR inhibitors	Adaptive feedback (insulin/IRS reactivation) and pathway cross-talk can limit durability	Suppressed PI3K/AKT signaling, often co-altered and activated with ARID1A loss, and can reduce HR; clinical combinations (olaparib + alpelisib) have shown signals of activity in HR proficiency and are mechanistically attractive in ARID1A contexts	FDA approved for PIK3CA-mutant breast cancer; combined with PARPi in ARID1A models; clinical development in combination strategies for ARID1A loss	[54,75,80]
8	Pictilisib					
9	Dactolisib					
10	Capivasertib					
11	Berzosertib	ATR inhibitors	Synergy with PARPi or replication stress agents; might overcome some HR restoration mechanisms but require clinical validation	Inhibit ATR-checkpoint signaling that ARID1A-deficient cells rely on for replication stress management; ARID1A deficiency increases dependence on ATR; ATR inhibitors are proposed and validated as synthetic–lethal partners in ARID1A-null models	Phase I/II trials; synthetic lethal strategy for ARID1A loss	[75,77,81,82]
12	Ceralasertib					
13	MK-2206	Allosteric AKT inhibitors	Parallel pathway activation, metabolic/epigenetic adaptations	PI3K inhibition sensitizes models to PARPi; clinical combinations (olaparib + alpelisib) have shown signals of activity in HR-proficient settings and are mechanistically attractive in ARID1A contexts	Phase III trials; proposed for ARID1A-deficient tumors	[54,77,80,83]
14	Ipatasertib					
15	Floxuridine (FUDR)	Pyrimidine analogue; thymidylate synthase inhibition causing DNA damage	Unknown clinical resistance patterns in ARID1A tumors; mechanism is DNA damage response (DDR) impairment dependent and might combine with DDR targeting agents	Thymidylate synthase inhibition causes DNA damage; ARID1A-deficient CRC cells were selectively sensitized to FUDR in vitro and in vivo, suggesting a non-PARPi therapeutic avenue	FDA-approved antineoplastic (regional hepatic infusion use); preclinical ARID1A-deficient CRC sensitivity demonstrated	[84]

## Data Availability

Not applicable.
